# Health Related Quality of Life in Iranian Patients With Irritable Bowel Syndrome: Reliability and Validity of the Persian Version of the IBS-QOL

**DOI:** 10.5812/ircmj.4605

**Published:** 2013-08-05

**Authors:** Peyman Jafari, Zahra Asadollahi, Maryam Moini, Mahdi Seyed Mirzaie

**Affiliations:** 1 Department of Biostatistics, Shiraz University of Medical Sciences, Shiraz, IR Iran; 2 Gastroenterology and Hepatology Research Center, Shiraz University of Medical Sciences, Shiraz, IR Iran; 3 Digestive Disease Research Center, Tehran University of Medical Sciences, Tehran, IR Iran; 4 Rafsanjan University of Medical Sciences, Rafsanjan, IR Iran

**Keywords:** Quality of Life, Irritable Bowel Syndrome, Questionnaires

## Abstract

**Background:**

Irritable bowel syndrome (IBS) is a gastrointestinal disease that is accompanied by pain, diarrhea, constipation and abdominal bloating. Hence, IBS has a major effect on patients’ quality of life.

**Objectives:**

The present study aimed to examine validity and reliability of the Persian version of the IBS-QOL questionnaire in Iran.

**Patients and Methods:**

The original 34 items of the IBS-QOL were translated from English into Persian through a structured procedure for the translation and cultural adaptation of the original English IBS-QOL into Persian. Ninety one patients who had GI symptoms but did not have any organic diseases (including 70 IBS patients diagnosed by Rome II criteria) were recruited from teaching hospitals Shiraz University in Iran and completed a Persian version of the IBS-QOL along with a Persian version of Room ΙΙ and IBS severity index (IBSSI).

**Results:**

Our findings showed that the IBS-QOL has excellent convergent and acceptable discriminant validity. All domains had Cronbach's alpha greater than 0.7 except health worry. Seventy patients who were diagnosed as IBS by the Room ΙΙ had significantly lower scores in the IBS-QOL than those who were not (FBD) (43.7 ± 20.1 vs. 73.4 ± 14.9, P < 0.01). Age, sex, education or marital status did not affect scores in measuring the quality of life.

**Conclusions:**

In conclusion, the Persian version of the IBS-QOL provided a well-defined measure of QOL in IBS patients with high validity and reliability that is an appropriate measure to use in further IBS clinical studies in Iran.

## 1. Background

IBS is a gastrointestinal disease that is accompanied by pain, diarrhea, constipation and abdominal bloating (1). Although IBS is a common disease, there are no obvious symptoms for diagnosis and it has been tried to create diagnostic criteria by combining symptoms (2). Diagnostic criteria used in the most diagnosis of IBS are Manning, Rome II and Rome I (3). Women are 2 or 3 times more exposed to IBS than men (4) The prevalence of this disease has been reported in different populations vary from 9 to 22 percent(5). The disease is one of the most common disorders in European countries (6) and known as the second reasons of workplace absence after cold in the United State (7). Moreover in the Asian countries the prevalence of IBS symptoms was reported low (8).

IBS is a chronic disease if patients are regularly monitored only 5 percent of them will be symptom -free after 5 years after the onset of the disease. Although it is not life threatening, its symptoms can be a major impact on patients’ quality of life and have considerable effect on different aspects of daily life. IBS has almost a hand in work, travel, nutrition, social and family relationships, sexual lifestyles, rest and ultimately affect the patient's way of life. Previous Researches show that the quality of life of patients with IBS compared to patients with other gastrointestinal diseases (such as reflux) or other chronic diseases such as asthma and migraine has a lower level (3, 9).

In recent years, studies to determine the prevalence of IBS in certain regions or groups in Iran have been performed. In these studies the prevalence of IBS was 5.6%, 4.2%, 11.8% in women, students, qashqai migrating nomads groups respectively (10-12). Considering all the above points, evaluation of IBS patients’ feeling is essential. The quality of life of patients with IBS is either evaluated by the general or specific questionnaires (9, 13). One of the few dedicated questionnaire measuring quality of life of patients with IBS is IBS-QOL questionnaire. This instrument was designed by Patrick and his colleagues that included 34 items and it covers 8 different scales of QOL.(14).This instrument has been translated into several languages (i.e. Dutch, Spanish, French, Chinese, Korean and Japanese) and it has shown good psychometric properties. (15-22).Considering the studies that have been done so far in Iran there has been no study in order to evaluate the QOL of IBS patients with technical and standard IBS-QOL questionnaire.

## 2. Objectives

The aim of this study is to measure the QOL of patients with IBS and more important to assess the validity and reliability of Persian version of IBS-QOL questionnaire and culturally adapted for the first time in Iran in order to provide a clear picture of condition of these patients’ quality of life and pay more attention to the problem they are encountering.

## 3. Patients and Methods

In the first step of study, an English version of the questionnaire based on Persian common terminology was translated into Persian by two professional translators independently. Consequently, a Persian version was developed after comparing the two translations. This Persian version was then independently backward translated into English for content comparison by an English specialist. The original and back-translated versions were compared to detect any misunderstanding s, mistranslations or inaccuracies. The final Persian language version of IBS-QOL questionnaire was then prepared and feedback obtained from gastrointestinal experts and also questions were modified based on Iranian culture. In this cross-sectional study sampling method is convenience. Patients who suffer from intestinal disorder referred to teaching hospitals of Shiraz University (Namazi and Saadi) and Shahid Motahari clinic between August 2008 and December 2010 were invited to participate. Patients’ participation criteria included persons who: were 18-65 years old; did not have any organic disease with symptoms during the study; get a minimum score of 75 on the IBSSI questionnaire; at least an overall elapsed time of six months from their intestinal disorder. All the participants gave written informed consent.

The instrument that was used to gather information contains four sections. The first part includes personal and social information such as age, sex, education level, marital status. The second part is related to the Persian version of the Rome ΙΙ modular questionnaire used to diagnose IBS. This instrument includes 4 main questions used to define IBS. Two or three of the following symptoms must be at least 3 weeks in the last 3 months: Relieved with defecation; onset associated with a change in frequency of stool; onset associated with a change in form (appearance) of stool (23). Moreover, considering the designed questionnaire, patients are categorized into 3 types according to IBS symptom patterns as follows: constipation dominant symptoms, diarrhea dominant symptoms and mixed symptoms. The third part consists of IBS Severity Index (IBSSI). This questionnaire contains 5 questions using the VAS scale: severity and duration of abdominal pain, severity of abdominal distension, dissatisfaction with bowel habits and interference with life in general quality of life. There’s a quantitative score ranging from 0 to 500 used to determine severity of symptoms with a score <75 defined patients in remission; between 75 and 175 mild IBS; between 176 and 300 moderate IBS; and > 301 severe IBS (24).

The last part includes the Persian version of the IBS-OQL questionnaire. The IBS-OQL consists of 34 items with 5 points response scales and 8 different subscales as follows: dysphoria (8 items), interference with activity (7 items), body image (4 items), health worry (3 items), food avoidance (3 items), social reaction (4 items), sexual concerns (2 items) and relationships (3 items).14 Raw scores are converted on the IBS-QOL into 0 to 100 points which higher values indicate better QOL. Information has been gathered based on face to face interviews by trained professionals.

### 3.1. Statistical Analysis

In this study in order to compare subgroup, parametric statistical tests have been used such as analysis of variance (Anova) or T test. Cronbach’s alpha was calculated to assess internal consistency. The criterion level for coefficient alpha was set at 0.7 or above (25). Convergent end discriminant validity was examined using spearman correlation. In order to consider validity of convergent, the value correlation of each item is calculated with its own scale and correlation coefficient of greater than 0.4 is regarded as an adequate evidence of convergent validity. Discriminant validity is evaluated by taking correlation of each item into account with other scales (26) discriminant validity works whenever a correlation between an item and its own scale is significantly higher than its correlation with the other scales (26). The overall score for the IBSSI and the scores for the 5 components at baseline were used to assess convergent validity of the IBS-QOL. All statistical analyses were carried out using the SPSS 15 and MedCalc.

## 4. Results

Table 1 shows demographic characteristics of patients. 

**Table 1.** Characteristics of the Study Patients (n = 91)

Characteristic	Number (%) of Patients
**Sex**	
Male	29 (68)
Female	62 (32)
Age (yrs, mean ± SD)	35 ±10
**Age groups**	
≤ 40	66 (73)
> 40	25 (27)
**Marital status**	
Single	13 (14)
Married	78 (86)
**Education**	
illiterate or Primary school	22 (24)
secondary or High school	49 (54)
University	20 (22)
**Diagnosis by Rome II criteria**	
Irritable bowel syndrome (IBS)	70 (77)
Other functional bowel disorders (FBD)	21 (23)
**IBS subtypes of bowel habit**	
constipation	27 (30)
diarrhea	34 (37)
Mixed	30 (33)
**Classification of symptom severity by IBSSI ** ^**a**^	
Mild	15 (17)
Moderate	35 (38)
Severe	41 (45)

^a^ Abbreviations: IBSSI, IBS severity index

The overall Cronbach’s alpha questionnaire 0.91 suggests that the questionnaire has high internal consistency. As indicated in Table 2, the value of Cronbach’s alpha coefficients obtained in all aspects of Persian version of IBS-QOL questionnaire is appropriate and represents the reliability of an acceptable questionnaire. Table 2 compares the mean scores on the IBS-QOL for mild, moderate, and severe IBS symptom. 

**Table 2.** Reliability for the Overall and Individual Scores on the IBS-QOL

Overall IBS-QOL and Subscales (number of items)	Mild (N = 15)	Moderate (N = 35)	High (N = 41)	Cronbach's alpha
**Overall scale**	75.2	55.9	37.0	0.91
**Dysphoria (8)**	77.0	47.1	28.9	0.90
**Interference with activity (7)**	75.4	59.0	37.5	0.77
**Body image (4)**	77.9	62.1	45.2	0.71
**Health worry (3)**	67.7	46.9	29.2	0.56
**Food avoidance (3)**	58.3	48.8	31.5	0.72
**Social reaction (4)**	69.1	51.4	38.4	0.70
**Sexual concerns (2)**	90.1	68.1	45.4	0.83
**Relationships (3)**	86.1	64.0	40.0	0.75

Based on age, sex, marital status and education level, Analysis showed that there’s no significant difference in overall QOL scores of patients (IBS-QOL). Although quality of life scores were higher in the younger age group than in the older group, this difference was not statistically significant. Overall quality of life scores of IBS patients with symptoms of diarrhea predominant bowel movements was less than other patients but this difference was not statistically significant. Analysis also confirms that the overall score of IBS-QOL has significant correlation with overall disease severity score on the IBSSI (p< 0.001 and r = -0.62) and its correlation was assessed with individual question on the IBSSI. The overall score of IBS-QOL is strongly correlated with abdominal distention questions, bowel movements and disease impact on the quality of life. Nonetheless, it is weakly correlated with abdominal pain (severity and duration) (Table 3). 

**Table 3.** Correlations Between Overall Score of the IBS-QOL and Overall and Individual Scores of the IBSSI

IBSSI	IBS-QOL	
	Correlation	Significance
**Overall**	-0.62	0.00
**Abdominal pain (severity)**	-0.21	0.04
**Abdominal pain (duration)**	-0.22	0.03
**Abdominal distension**	-0.43	0.00
**Bowel movement**	-0.45	0.00
**Quality of life**	-0.58	0.00

Table 4 represents percentage of convergent and discriminant validity. Correlation of each item with its own scale was higher than 0.4. Convergent validity was 100 % in all the scales. All the scaling success rates in the assessment of discriminant validity were higher than 75% except for interference with activity. 

**Table 4.** Item Scaling Tests: Convergent and Discriminant Validity for IBS-QOL Scales

Subscales (number of items)	Convergent validity ^a^		Discriminant validity ^b^	
	Range of correlation	Scaling success (percent)	Range of correlation	Scaling success (percent)
**Dysphoria (8)**	0.64-0.84	8/8 (100)	0.30-0.69	47/56 (84)
**Interference with activity (7)**	0.56-0.74	7/7 (100)	0.16-0.67	30/49 (61)
**Body image (4)**	0.61-0.80	4/4 (100)	0.30-0.67	21/28 (75)
**Health worry (3)**	0.71-0.77	3/3 (100)	0.28-0.57	20/21 (95)
**Food avoidance (3)**	0.72-0.89	3/3 (100)	0.23-0.53	21/21 (100)
**Social reaction (4)**	0.60-0.74	4/4 (100)	0.22-0.62	24/28 (86)
**Sexual concerns (2)**	0.91-0.92	2/2 (100)	0.41-0.59	14/14(100)
**Relationships (3)**	0.76-0.85	3/3 (100)	0.37-0.74	19/21 (90)

^a^Number of correlation between items and scale corrected for overlap ≥ 0.4/ total number of convergent validity tests

^b^Number of convergent correlations significantly higher than discriminant correlations/ Total number of correlations

There was significant difference in the overall scores or each of individual score on the IBS-QOL between patients who were diagnosed as IBS by the Room ΙΙ and 21 other patients who were not (FBD) in the participants.

## 5. Discussion

This study showed that the Persian version of the IBS-QOL is a reliable and valid instrument. All scales exceeded the minimum reliability criterion of a Cronbach’s α coefficient over 0.70. In a study that has been conducted by Patrick in the original version showed to have Cronbach’s alpha score of 0.95 and mean coefficient of 0.77 in different scales.( 14 ) as it is understood, there is not much of a difference between these coefficients and the coefficients of this study. Moreover, based on age, sex, marital status and education level, Analysis showed that there has not been any significant difference between the total score and each of individual on the IBS-QOL. Our results in the present study were quit similar to that in other studies( 14 , 19 , 20 )Analysis also confirms that overall score on the IBS-QOL is strongly correlated with IBS Severity Index (IBSSI). Besides, this result was in agreement with the Japanese study ( 19 ). Patients who were diagnosed as IBS by the Room ΙΙ showed a lower overall score on the IBS-QOL than those who were not diagnosed by the Room ΙΙ and this difference was not significant in Japanese version ( 19 ). Our study as like as other studies did not confirm that there are significant differences in the QOL score among subtypes of bowel movement ( 14 , 19 ). As expected, patients in severe conditions have IBS-QOL scores significantly lower than patients with mild and moderate. The lowest mean scores on IBS-QOL concerns with food avoidance and health worry scales in all 3 groups and the highest score deals with sexual concerns (see Table 2). 

Our findings suggest that the Persian version of the IBS-QOL has excellent convergent and acceptable discriminant validity (Table 4). The lowest score of the IBS-QOL questionnaire in Iranian IBS patients is the health worry scale and this score is low in comparison with other countries. This difference is due to Iran’s cultural and social differences with other countries in reaction to symptoms of the disease and the increase of patients’ worry is due to the more existence of disease. The highest score is referred to sexual scale which means IBS does not affect sexual life in some patients. However, the cause of this could be related to the fact that Iranians don’t express their sexual activities to someone in comparison with people in the western countries. Sexual problems may well exist, but cultural factors (believes, religion and social milieu) may inhibit questionnaire respondents from admitting to this difficulties. Iranian patients with IBS as compared with patients in the U.S and Japan had lower quality of life score in all domains. Although part of this could due to cultural and social differences of Iran and the two other countries but it is necessary to mention the fact that 83 percent of participants have moderate and severe severity disease whereas in U.S and Japan study only 63 and 68 percent of participants have moderate and severe severity disease ( 14 , 19 ). The mean overall score of the IBS-QOL in Iranian patients also showed a similar result with that measured by the China version of the IBS-QOL. Nevertheless, the mean individual scores of dysphoria (35vs. 45), health worry (34 vs. 50), food avoidance (37 vs.49), sexual concerns (54 vs. 38) on the Persian version were over approximately 12-point lower except sexual concerns ( 27 ). In conclusion, the Persian version of the IBS-QOL provided a well-defined measure of QOL in IBS patients with high validity and reliability that is an appropriate measure to use in further IBS clinical studies in Iran. 
